# Editorial

**DOI:** 10.1093/emph/eos001

**Published:** 2012-08-30

**Authors:** Stephen C. Stearns

**Affiliations:** New Haven, CT, USA

When a new journal was founded 25 years ago, its editor wrote [1]: ‘The arguments against new journals are many, and some are convincing. New journals often signal the arrival of a new specialty; their rate of origin roughly measures the rate of fragmentation of a science … . Because the literature is already too large and too fragmented for any one person to be able to keep up to date, adding a new journal just makes an already bad situation worse … . Nevertheless, with this issue a new journal is born, and the reasons for bringing it into existence had better be good. They are.’ My reasoning then also applies today.

*Evolution, Medicine, and Public Health* (*EMPH)* has been founded to integrate the emergence of evolutionary thinking in many parts of medical science. Like physics and chemistry, evolution is a basic science that permeates medicine and helps to generate explanations for everything we encounter in it. Evolutionary medicine therefore includes any area in which evolutionary thought sheds light on medical and epidemiological issues. Those areas are diverse; they include many traditional disciplines.

What will this journal publish? The relevant subjects include at least these [2]: the origin, maintenance and medical significance of human genetic variation; mismatches to modernity, with implications for autoimmune disease, diabetes, obesity and cancer; special features of human reproductive biology, including menopause; broader features of mammalian reproductive biology, including evolutionary conflicts of interest between parents, between parents and offspring, and among offspring, with implications for pre-eclampsia and pregnancy-related diabetes; the evolution of aging and how it explains our susceptibility to degenerative disease; cancer as a somatic evolutionary process in which resistance to chemotherapy and metastasis results from natural selection operating on clonal genetic variation; pathogen evolution—especially virulence, drug resistance, and evasion and suppression of the immune system; pathogen evolution to emerge as agents of new diseases; the evolution of vectors that transmit disease; insights from comparisons with other species; and ideas on how best to teach evolution in medical curricula. Articles on all these subjects will get serious consideration in *EMPH*.

There are also important research questions to be answered [2]: Can antimicrobial therapies and anti-vector campaigns be made evolution-proof? Will imperfect vaccines increase virulence? Can evolutionary insights improve cancer therapy? Can parasitic worms inspire therapies for auto-immune diseases? Will molecular phylogenetics identify the ancestral states of metastases and thus suggest new tools for early cancer detection? Can we exploit our natural defenses to attack cancer? Will understanding early–late life history tradeoffs suggest treatments for aging? What is the role of parent-of-origin genetic imprinting in mental disease? Can evolved mechanisms of intra-genomic conflict be used therapeutically? Can we switch the host–pathogen relationship from resistance to tolerance and, if so, at what cost? Answers to such questions belong on the pages of this new journal.

The publication of new research is *EMPH*’s highest priority; the core unit is, for the time being, the original research article. There are also roles here for reviews, commentaries on hypotheses and controversies, book reviews, case studies, and correspondence about recently published articles. Formats for each are available on our website: http://www.oxfordjournals.org/our_journals/emph/for_authors/scope_and_purpose.html.

Open access is particularly appropriate for a new interdisciplinary journal, whose readers often will not have access to subscription journals in all the fields covered and where libraries often will not have funds for new subscriptions. For this open access journal, we have negotiated a package of fees and waivers that favors authors as much as possible while ensuring financial stability and publisher support. In return for the opportunity to publish in *EMPH*, we offer authors the credibility of rigorous referees; the visibility and prestige of a prominent publisher; and the stability, distinction and wisdom provided by 17 members of the Board of the Evolution, Medicine, and Public Health Foundation, which owns the journal, and by the 89 associate editors who make up the Editorial Board.

Evolutionary medicine can help to reduce suffering and save lives. It is also full of new ideas, unexpected connections and scientific surprises. There is room here for research on all of that.
